# Identification of genomic insertion and flanking sequences of the transgenic drought-tolerant maize line “SbSNAC1-382” using the single-molecule real-time (SMRT) sequencing method

**DOI:** 10.1371/journal.pone.0226455

**Published:** 2020-04-10

**Authors:** Tingru Zeng, Dengfeng Zhang, Yongxiang Li, Chunhui Li, Xuyang Liu, Yunsu Shi, Yanchun Song, Yu Li, Tianyu Wang

**Affiliations:** Institute of Crop Sciences, Chinese Academy of Agricultural Sciences, Beijing, China; University of Helsinki, FINLAND

## Abstract

Safety assessment of genetically modified (GM) crops is crucial at the product-development phase before GM crops are placed on the market. Determining characteristics of sequences flanking exogenous insertion sequences is essential for the safety assessment and marketing of transgenic crops. In this study, we used genome walking and whole-genome sequencing (WGS) to identify the flanking sequence characteristics of the *SbSNAC1* transgenic drought-tolerant maize line “SbSNAC1-382”, but both of the two methods failed. Then, we constructed a genomic fosmid library of the transgenic maize line, which contained 4.18×10^5^ clones with an average insertion fragment of 35 kb, covering 5.85 times the maize genome. Subsequently, three positive clones were screened by pairs of specific primers, and one of the three positive clones was sequenced by using single-molecule real-time (SMRT) sequencing technology. More than 1.95 Gb sequence data (~10^5^× coverage) for the sequenced clone were generated. The junction reads mapped to the boundaries of T-DNA, and the flanking sequences in the transgenic line were identified by comparing all sequencing reads with the maize reference genome and the sequence of the transgenic vector. Furthermore, the putative insertion loci and flanking sequences were confirmed by PCR amplification and Sanger sequencing. The results indicated that two copies of the exogenous T-DNA fragments were inserted at the same genomic site, and the exogenous T-DNA fragments were integrated at the position of Chromosome 5 from 177155650 to 177155696 in the transgenic line 382. In this study, we demonstrated the successful application of the SMRT technology for the characterization of genomic insertion and flanking sequences.

## Introduction

Since genetically modified (GM) crops were first introduced in the US in the mid-1990s, they have become widely adopted by growers of many countries in the world [1]. In 2017 alone, 189.8 million hectares of GM crops were planted worldwide [2]. It is an international consensus that GM crops may be commercialized after they are indicated to be safe. As a result, extensive testing and comprehensive analyses of transgenic lines with excellent objective traits are necessary for biosafety assessment before the lines may be approved and entering the market. Among these methods, molecular characterization of GM crops at the chromosomal level, including insertion sequences, sites, copy numbers and flanking sequences, is essential for the safety assessment and specific detection of GM crops [3, 4]. Furthermore, identifying T-DNA flanking sequences of GM crops and developing specific detection methods are useful for breeding programs and important for bio-risk management to ensure food, feed and environmental safety [5, 6].

Traditionally, exogenous fragments flanking sequences of transgenic plants are obtained by various PCR-based methods according to the T-DNA sequence information [7–10]. Among these methods, thermal asymmetric interlaced PCR (TAIL-PCR) and genome walking are often used to isolate and clone T-DNA flanking sequences [9, 10]. For example, by using the TAIL-PCR method by sequencing, several T-DNA flanking sequences were identified and characterized in transgenic maize [11], soybean [12], cotton [13], and alfalfa [14]. However, these PCR-based methods are labor-intensive and expensive.

With the emergence and rapid development of the next-generation sequencing (NGS) technology over the past several years, molecular characterization of insert locations, copy numbers, integrity, and stability of transgenic crops can be implemented in a relatively short time and at acceptable cost [15]. To date, a number of flanking sequences of exogenous genes in GM plants, such as *Arabidopsis* [16], rice [17], soybean [6], and maize [18], have been identified by NGS.

Taken together, both the PCR-based method and the NGS technology enabled us to successfully characterize both single and stacked transgenic events [15]. However, these approaches have difficulty identifying all insertion loci and their flanking sequences of transgenic events with complex genome sequences or intricate modifications or rearrangements of exogenous fragments [6, 19]. For instance, maize has a larger genome and more repetitive sequences than soybean, cotton and rice, and it is difficult to identify flanking sequences of inserted genes [20]. In addition, transgenic events of maize often contain a part of or the entire vector backbone. In other cases, a partial copy of T-DNA inserts and connections takes place outside given expected boundaries [21, 22]. For the acquisition of flanking sequences integrated into larger genomes and complex insertion fragments, accurate flanking sequences can often be found by constructing DNA libraries. Turning genomes into countless fragments by physical or biological means and then cloned in fosmid or BAC vectors were a mainstay of genome projects during the Sanger-based sequencing era [23, 24]. Compared with other libraries, fosmid libraries have the advantages of short cloned fragments (approximately 40 kb), single-copy insertion and ease of generation [25]. Because inserts in the fosmid libraries are generated randomly by the ultrasonic method, rather than by enzymatic digestion, inserts in the fosmid libraries can avoid potential clone biases. This method is suitable for physical mapping, gene cloning and chromosomal mapping of gene fragments [6, 26]. Recently developed single molecule-based NGS technology generates longer reads (20 kb) at increased coverage depth and is particularly important in resolving the challenges in the characterization of transgenic events with insert locations in repetitive and low-complexity regions of a genome [27]. As a result, using fosmid libraries and the single molecule-based NGS technology might be suitable for identifying T-DNA flanking sequences of transgenic lines of crops with complex genomes such as maize or intricate modifications or rearrangements of exogenous fragments.

Recently, we developed one transgenic line, “SbSNAC1-382”, by overexpression of *SbSNAC1* from sorghum, which conferred drought tolerance without a cost of crop productivity under well-watered conditions. Southern blotting confirmed that SbSNAC1-382 was a two-copy insertion event, and the two copies might be inserted at the same location in the genome. To obtain the flanking sequence of the target gene of the transgenic maize event, after the failure of the genome walking method and the whole genome sequencing method, the single-molecule real-time sequencing was used to identify the accurate flanking sequences of the inserted gene. Molecular characterization of drought-tolerant transgenic maize at the nucleic acid level may provide precise information for regulatory submissions and facilitate utilization of the line in future breeding programs.

## Materials and methods

### Plant materials

*SbSNAC1* with *Bst*EⅡ and *Nco*I enzymatic restriction sites were recombined into the pCAMBIA3301 vector under control of the cauliflower mosaic virus (CaMV) 35 S promoter, resulting in 35S::*SbSNAC1* constructs (S1 Fig). The constructed vector was transferred into the maize hybrid, HiII, by the *Agrobacterium*-mediated method. Positive transgenic events were backcrossed with the inbred line “Zheng58” for six generations, and the resulting “SbSNAC1-382” was used in subsequent flanking sequence identification.

### DNA isolation and Southern blot analysis

Genomic DNA from leaf samples of the transgenic event and the non-transgenic control was isolated using the CTAB method [28].

Thirty micrograms of the genomic DNA from the transgenic event and the non-transgenic control were digested with the restriction enzymes *BstE*II and *Nco*I overnight. The resolved genomic DNA was then transferred to positively charged nylon membranes (Hybond-N^+^, Amersham Pharmacia Biotech) using the model 785 vacuum blotter system (Bio-Rad). The *Bar* amplified fragments (Table 1) were labeled by the DIG high primer DNA labeling (Roche, Cat. No. 11585614910) and purified using the high-purity PCR product purification kit (Roche, No.11732668001). The DNA blots were pre-hybridized at 42°C for 1 h in DIG easy hybgranules and then hybridized to denatured DIG-labeled probes for 20 h. The blots were then washed twice with 2×SSC and 0. 1% (w/v) SDS for 15 min each and washed twice with 1×SSC and 0.1% (w/v) SDS for 15 min each. Immunological detection of the probes was performed in accordance with the manufacturer’s instructions for the DIG high primer DNA labeling and detection starter kit II.

**Table 1:** Primers used in this study.

Primer	Sequence (5’-3’)
Bar F	GAAGTCCAGCTGCCAGAAAC
Bar R	GTCTGCACCATCGTCAACC
SbNACS3	GACCGCAAGTACCCAAACGG
SbNACA3	CACCCAGTCATCCAGCCTGAG
SbNACS4	GGGACCGCAAGTACCCAAACG
SbNACA4	GCTGCGCTTCTCGCTCCTCT
NosF1	GAATCCTGTTGCCGGTCTTG
NosR1	TTATCCTAGTTTGCGCGCTA
35F1	GCTCCTACAAATGCCATCATTGC
35R1	GATAGTGGGATTGTGCGTCATCCC
zsp1	TATCCCTGGCTCGTCGCCGA
zsp2	AGGGCTTCAAGAGCGTGGTCGCT
zsp3	CCGTCACCGAGATTTGACTCGAGTTTC
YZP1	AGAATCATACACCAGTAACAAGCC
YZP2	CAGTACATTAAAAACGTCCGCA
YZP3	ACTAAAATCCAGATCCCCCGAA
YZP4	TTCACACAAGGAAACAGCTATGA
YZP5	CGATTAAGTTGGGTAACGCCA
YZP6	CTTCGCAAGACCCTTCCTCT
YZP7	TCCCTCTCCCTCCTCATCAC
YZP8	AGATTTTCTTCTTGTCATTGGG
YZP9	CTAGAGCAGCTTGAGCTTGG
V1	GGTTTCGCTCATGTGTTGAGC
G1	AGTGCACATTGCAATCCTACAAG
G2	CCTAAGTTCATGCAACTAGAGGTTTCA

### Genome walking method

The 5' flanking sequence of the insertion sequence was obtained by the genome walking kit according to the manufacturer’s instructions (TaKaRa, Dalian, China). The random primers were provided by the genome walking kit, and the specific primers were designed based on theoretical insertion sequences (first round zsp1; second round zsp2; third round zsp3, Table 1). The specific PCR products were gel-purified by using the DNA Gel Extraction Kit (Axygen, USA), cloned into the pMD-18 vector system (Takara), and then sequenced by the Shanghai Sangon Company.

### Whole genome sequencing

A total of 1.5 μg DNA per sample was used as input material for the DNA sample preparations. Sequencing libraries were generated by using the Truseq Nano DNA HT Sample preparation Kit (Illumina USA) following the manufacturer’s recommendations, and index codes were added to attribute sequences to each sample. Briefly, the DNA sample was fragmented by sonication to a size of 350 bp, and then DNA fragments were end-polished, A-tailed, and ligated with the full-length adapter for Illumina sequencing with further PCR amplification. Finally, PCR products were purified (AMPure XP system), and libraries were analyzed for size distribution by an Agilent 2100 Bioanalyzer and quantified using real-time PCR. These libraries were sequenced by the Illumina HiSeq4000 platform, and 150-bp paired-end reads were generated with an insert size of approximately 350 bp.

### Construction and screening of the fosmid library

DNA was broken into fragments by ultrasonication and separated by pulsed field gel electrophoresis (PFGE). DNA fragments from 38–48 kb were recovered, and the recovered DNA fragments were end-repaired to be blunt and were 5’-phosphorylated. The fosmid library was constructed with the Copy Control^™^ HTP Fosmid Library Production Kit (Epicenter, USA) using the pCC2FOS^™^ Vector and EPI300-T1^R^ plating cells.

Three pairs of vector-specific primers were designed to screen positive clones from the fosmid library (SbNACS3/SbNACA3; SbNACS4/SbNACA4; Bar F/Bar R, Table 1). In the initial screening of the library, three pairs of primers were used to detect the library. After a positive clone was identified, it was diluted 2 × 10^6^ times with LB liquid media and plated on LB solid medium. Then monoclones were picked and subjected to colony PCR. The Colony PCR procedure was as follows: 95°C for 5 min; 95°C for 30 s; 60°C for 30 s; 72°C for 30 s; and a final extension at 72°C for 5 min; 32 cycles were employed.

### Single-molecule real-time sequencing

Ten micrograms of plasmid was extracted and purified by amplification of the positive colony selected in the previous step. The PacBio libraries were constructed using a plasmid that was mechanically sheared to a size of ~22 kb using Covaris g-TUBE (Covaris, Inc., Woburn, MA) according the manufacturer’s instructions. PacBio SMRTbell libraries were prepared by ligation of hairpin adapters at both ends of the DNA fragment using the PacBio DNA template preparation kit 2.0 for SMRT sequencing on the PacBio RS II machine (Pacific Biosciences of California, Inc., Menlo Park, CA). A bluepippin preparation system (SAGE Science, Beverly, MA) was used to enrich more than 7 kb fragments in the library. Then, the quality of the library was tested by the Agilent Bioanalyzer 2100 kit (Agilent Technology, Inc., Santa Clara, CA). Sequencing was performed on the PacBio RS II instrument according to the manufacturer’s recommendations.

## Results

### Southern blot analysis

**Fig 1:**
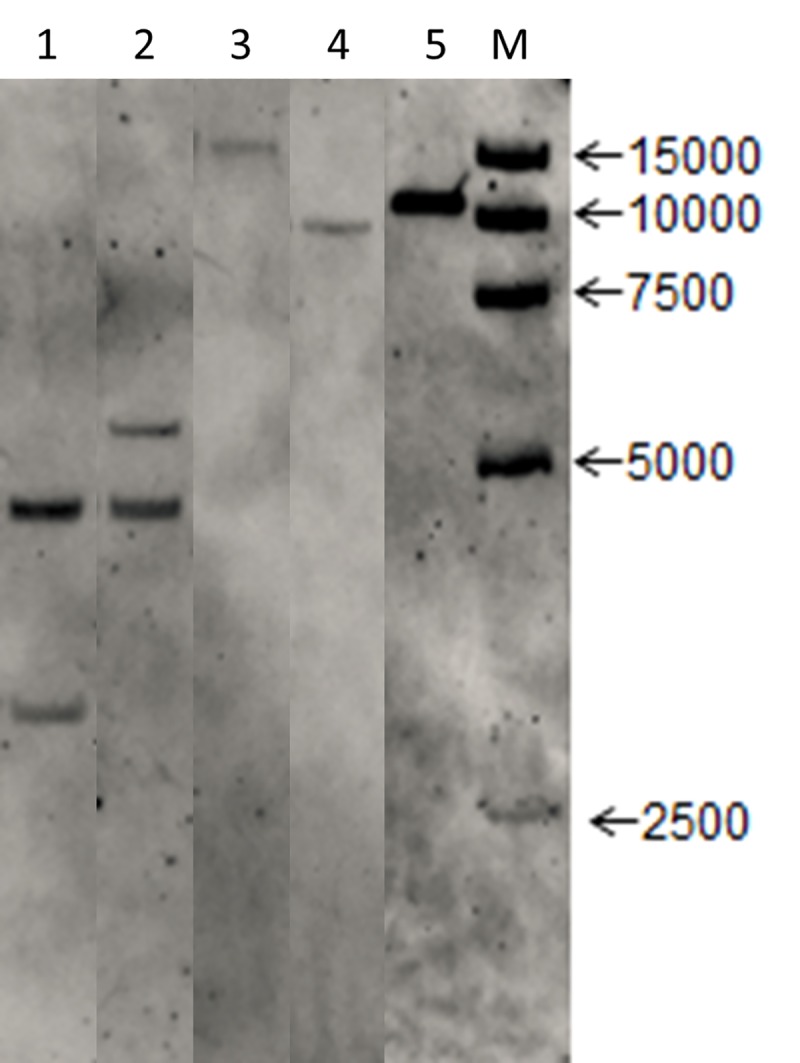
Southern blot analysis of the SbSNAC1-382 line. Lanes 1 to 4, digested DNA of the transgenic line by *Hind*Ⅲ, *Eco*RI, *Bgl*II and *Dra*I, respectively; lane 5, digested plasmids as positive controls; M, marker.

To determine the copy number of the transgenic event, a Southern blot analysis was performed by using probes designed to hybridize the *Bar* gene in the T-DNA sequences. The results showed that the transgenic line had two copies of insertion of the exogenous sequences when the restriction endonucleases *Hind*III and *Eco*RI were used to digest the DNA of the transgenic line (Fig 1). On the other hand, only one band was observed when the DNA of the transgenic line was digested with *Bgl*II and *Dra*I, for which there are no restriction sites in the insertion sequences (Fig 1). As a result, there might be two copies of insertion sequences at the same genomic location of the transgenic maize line.

### Genome walking for detecting flanking sequences

**Fig 2:**
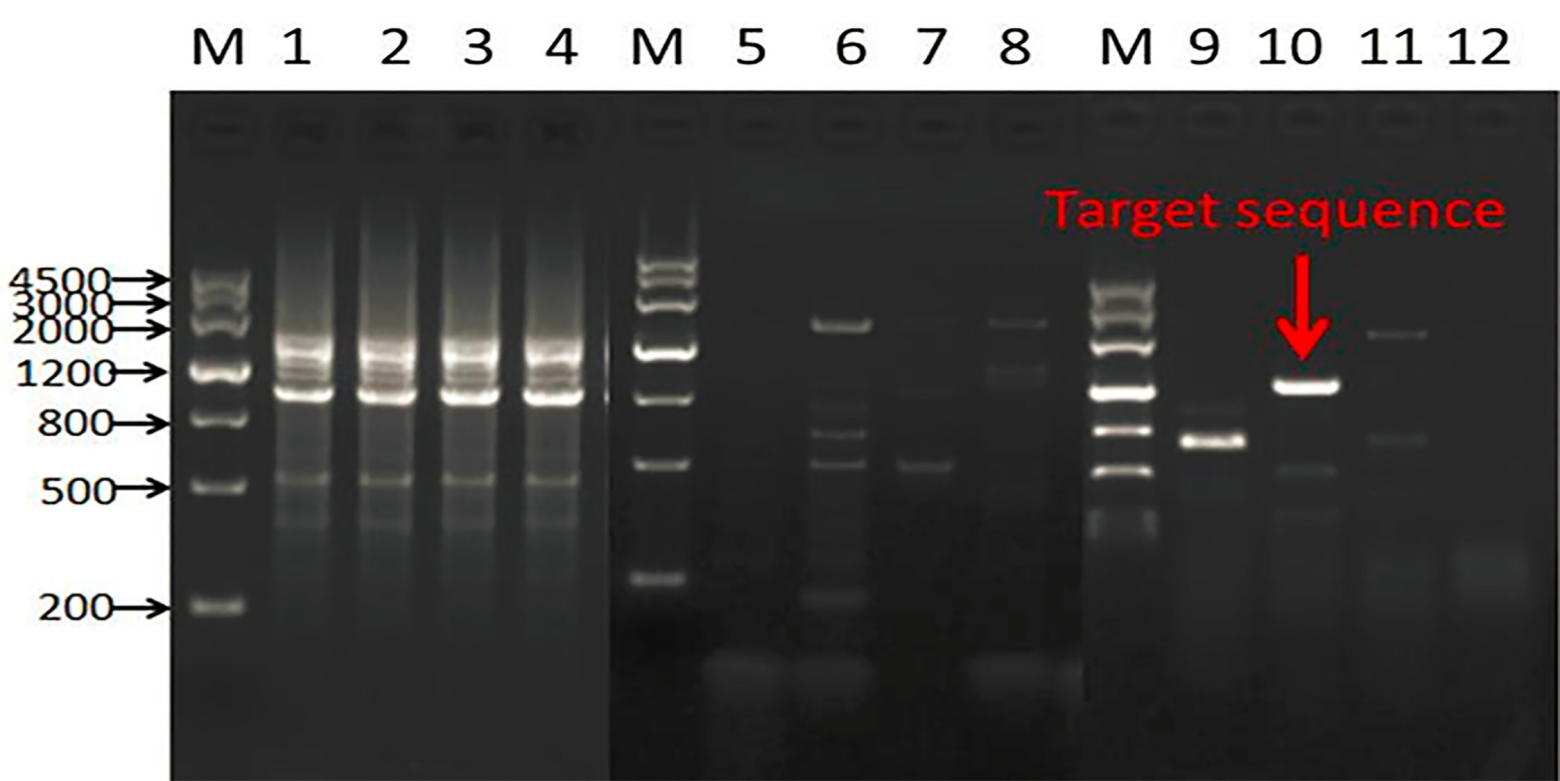
Genome walking results for the 5’ flanking sequence. Lanes 1–4 are the first amplification results of specific primer zsp1 and degenerate primer AP1-AP4, respectively; lanes 5–8 are the second amplification results of specific primer zsp2 and degenerate primer AP1-AP4, respectively; lanes 9–12 are the third amplification results of specific primer zsp3 and degenerate primer AP1-AP4, respectively; M: marker.

Three nested specific primers (zsp1, zsp2 and zsp3) were designed according to the sequences adjacent to the T-DNA left border. According to the instructions of the genome walking kit (Takara-Bio, Dalian, China), with nested specific primers and four degenerate primers, three rounds of nested PCR were completed, and a specific band was obtained (lane 10 of Fig 2). The sequencing results demonstrated that the specific PCR fragment contained 1227 bp in length. By aligning with the maize genome sequence of B73 on Maize GDB (www.maizegdb.org) and the T-DNA sequence, it was showed that the fragment was made up of 932 bp of non-insert DNA and 295 bp of insert DNA. As expected, the 295-bp inserted DNA was identical to the sequence adjacent to the T-DNA left border. The 932 bp of non-insert DNA was identical to the maize genome sequence, which is located between 177155650 and 177156582 bp on Chromosome 5. However, the flanking sequence adjacent to the T-DNA right border could not be identified with multiple nested specific primers and degenerate primers using the same method.

### WGS for detecting flanking sequences

We attempted to employ the WGS technique to identify flanking sequences on both sides of the insertion sequence. The libraries were sequenced by the Illumina HiSeq4000 platform, and 150-bp paired-end reads were generated with an insert size of approximately 350 bp. After quality control processing, a total of 144.6 billion clean reads for the transgenic line were obtained (Table 2). Among the reads, 96.77% of them could be mapped to the reference genome, accounting for ~ 64.57× coverage of the maize genome. Furthermore, approximately 93.66% of the genome had at least one fold coverage, and 88.57% had at least four fold coverage. Therefore, the above data indicate that the quality of sequencing met the requirements of analysis.

**Table 2:** Summary of sequence data of WGS.

Index	Value
Clean reads (bp)	144,595,902,900
Q20 (%)	> 90
Q30 (%)	> 85
Mapped reads (bp)	932, 861, 603
Total reads (bp)	963, 972, 686
Mapping rate (%)	96. 77
Average depth (X)	64.57
Coverage at least 1X (%)	93.66
Coverage at least 4X (%)	88.57

To identify flanking sequences of putative insertion sites of exogenous fragments, all clean reads were mapped to the sequence of the *pCAMBIA3301-SbSNAC1* vector and the maize reference genome. The putative flanking sequence of the SbSNAC1-382 line was characterized based on junction reads in which one end mapped to the vector sequence and the other end mapped to the maize genome. After detailed analysis was performed, five putative flanking sequences were found. One of the five possible flanking sequences was consistent with genome walking results. The total length of the fragment was 263 bp. The 150-bp DNA sequence was identical to the sequence adjacent to the T-DNA left border, and the 113-bp DNA sequence was identical to the maize genome. Unfortunately, the other four putative flanking sequences were not confirmed by the PCR results. As a result, the flanking sequence adjacent to the T-DNA right border of the SbSNAC1-382 line was still not identified by using the WGS technology.

### Fosmid library construction and positive clone screening

To identify the flanking sequence adjacent to the T-DNA right border of the SbSNAC1-382 line, we constructed a fosmid library of the SbSNAC1-382 line (Takara, Dalian, China) with a recombination rate of 100% (S2 Fig). The original library was diluted, and the number of colonies was counted. The library contained approximately 4.18× 10^5^ clones, and the average length of the inserted fragments was approximately 35 kb, which could achieve 5.85 times the maize genome coverage. According to the Clarke-Carbon formula [29], the probability of screening any gene or sequence from the constructed library was 99. 71%.

To screen the target clone from the fosmid library, five pairs of primers were designed (SbNACS3/SbNACA3; SbNACS4/SbNACA4; Bar F/Bar R, NosF1/NosR1, 35F1/35R1, Table 1) according to the T-DNA sequences. According to the results obtained by PCR methods, three positive clones were identified and stored for single-molecule real-time sequencing.

### Single-molecule real-time sequencing and Sanger sequencing

One of the three positive clones screened from the fosmid library was selected for sequencing. After the processing of quality control, yielding a total of 1.95 Gb of data, the average reading length of original reads was 19390 bp, and N50 was 28065 bp; the average reading length of subreads was 9509 bp, and N50 was 12507 bp (Table 3).

**Table 3:** Statistics of single-molecule real-time sequencing for plasmid DNA.

Index	Value
Polymerase read bases (bp)	1,949,620,057
Number of polymerase reads	100,544
Post filter mean read length (bp)	19,390
Polymerase read N50 (bp)	28,065
Polymerase read quality	0.83
Mean subread length (bp)	9,509
Subreads N50 (bp)	12,507
Number of subreads	204,500

**Fig 3:**
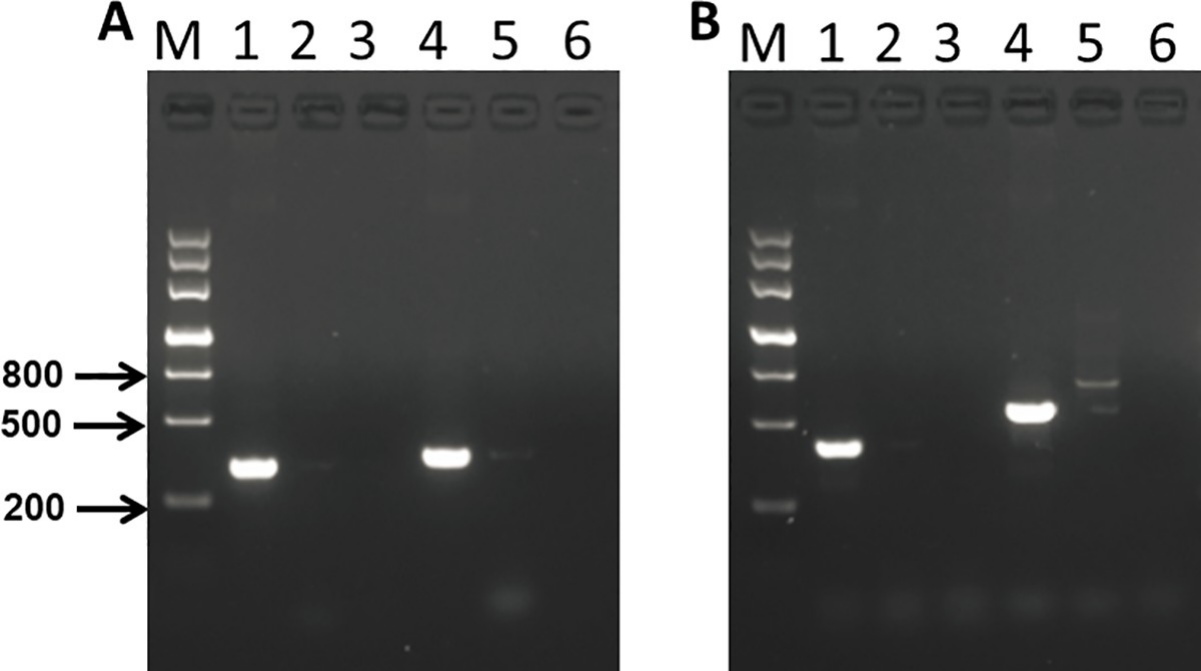
PCR validation of transgenic insertion sites. (A) PCR verification of the5' end of the inserted sequence. 1, 2, 3 and 4, 5, 6 primer YZP1/YZP2 and YZP1/YZP3 amplified in the transgenic line, negative control Zheng58, negative control of water, respectively. M: marker. (B) PCR verification of the 3' end of the inserted sequence. 1, 2, 3 and 4, 5, 6 primer V1/G1 and V1/G2 amplified in the transgenic line, negative control Zheng58, negative control of water, respectively.

**Fig 4:**
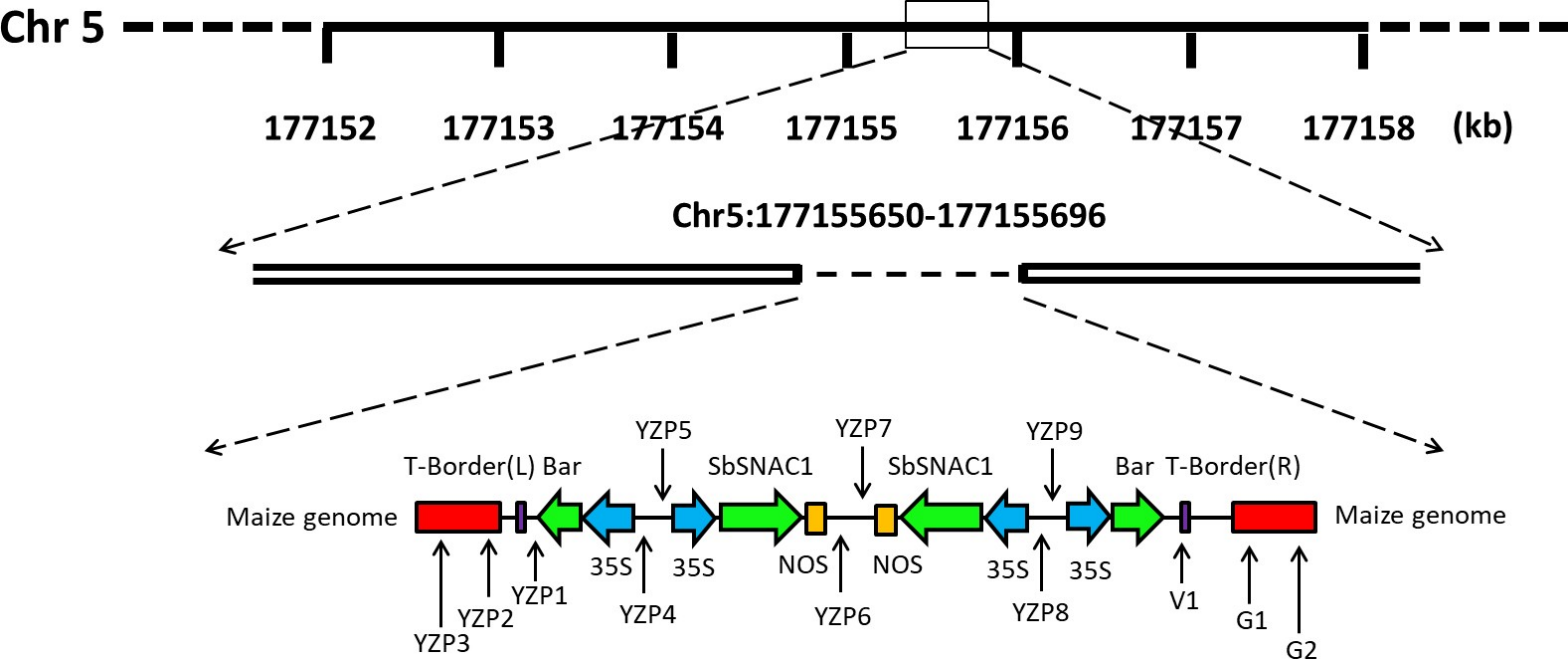
Schematic diagram of insertion loci and flanking sequences of SbSNAC1-382. The numbers under the line of Chr. 5 indicates physical positions on the chromosome. The arrows indicate the position of the validation primer.

To determine the hypothetical insertion sites of exogenous fragments, we constructed a local BLAST data library of single-molecule real-time sequencing data. According to the BLAST results of T-DNA sequences with the local data library, the Southern blot results confirmed that the exogenous sequences were composed of two copies of the *SbSNAC1* gene and the *bar* gene at the same maize genomic location, and the flanking sequences of both the right border and the left border were identified. To confirm the flanking sequences of the SbSNAC1-382 transgenic line, specific PCR primers were designed based on the putative genomic sequences and the insertion sequences. When using primer pairs with one primer annealing within putative flanking sequences (YZP2, YZP3, G1, and G2, Table 1) and the other annealing to the insertion sequence (YZP1 and V1, Table 1), gel electrophoresis revealed that PCR analyses of primer pairs (YZP1/YZP2, YZP1/YZP3, V1/G1 and V1/G2, Table 1) generated products with a single band in the transgenic event 382, while no correct product could be detected from the non-transgenic control of Zheng58 or the negative control of water (Fig 3). In addition, YZP3/G2 (Table 1) primers were used to amplify the whole length of the inserted sequence, and Sanger sequencing of the PCR products showed that the sequence was largely the same as that of the single-molecule sequencing, except for a few bases. Therefore, the exogenous sequence of the SbSNAC1-382 line was integrated at the physical position of Chr. 5: from 177,155,650 to 177,155,696 with a 46-bp deletion (Fig 4). Furthermore, the exogenous fragment was inserted into the intergenic region of the maize genome, and no functional genes were interrupted by the inserted sequence. To verify the results of single-molecule sequencing, we designed a series of primers on the insertion sequence (YZP2/YZP5, YZP4/YZP7, YZP6/YZP9 and YZP8/G1, Table 1). The PCR products obtained by these primers were sequenced and compared with the results of single-molecule sequencing. The two sequencing results were similar, suggesting that single-molecule sequencing had a high accuracy. Further analysis of the structure of the insertion sequence revealed that the exogenous sequence contained two insertion sequences with tandem repetition and opposite directions. Because the restriction endonucleases *Hind*III and *Eco*RI are between *bar* and *SbSNAC1*, there would be two bands after digestion with these two endonucleases. Meanwhile, for the genome digested with *Dra*I and *Bgl*Ⅱ with no sites in the insertion sequence, there was only a single band in the Southern blot results. The special structure of the insertion sequence explained the results of Southern blot analysis. Because of the special structure of the insertion sequence, neither the Sanger sequencing method nor the second-generation sequencing method could obtain the cloned sequence.

## Discussion

Detailed molecular characteristics of flanking sequences of insertions play an important role in the safety assessment of genetically modified crops [30]. In prior studies, PCR-based methods, such as tail-PCR and genome walking, have been used to determine the locations of integration sites and junction sequences between exogenous sequences and the host genome [9, 31]. With the continuous improvement of technology, the flanking sequence of single T-DNA copy insertion in transgenic lines can be obtained quickly and inexpensively by these PCR-based methods. For example, Ruslan et al. designed a PST (palindrome sequence targeting) primer to amplify the promoter sequence of the target gene quickly and efficiently [32]. Dietrich et al. amplified the 5’ flanking sequence of the insertion sequence of 75 *Mu* maize mutant lines based on the PCR method, but the flanking sequences of 20% of the lines could not be obtained by this method in their study [33]. These PCR-based methods may not work well if the deletion, modification or rearrangement occurred in exogenous insertion sequences. On the other hand, a high level of duplication or repetitive genome sequences adjacent to the exogenous fragment insertion location might increase the difficulty of identifying the flanking sequences. The maize genome size is approximately 2.3–2.5 Gb, and nearly 85% of the maize genome is composed of hundreds of families of transposable elements dispersed unevenly across the whole genome [34, 35]. In our research, the genome walking method was also used to amplify the flanking sequence of the insertion sequences, but only one end of the flanking sequence was identified due to the complex structure of the insertion sequences. As a result, using PCR-based methods to identify the flanking sequences of complex exogenous fragments of transgenic lines might be challenging in the maize genome.

With the emergence and development of high-throughput next-generation sequencing (NGS) technology, the cost of whole genome sequencing has been greatly reduced (Table 4). The NGS technology has been widely used in different species to discover such features as structural variation and rearrangements [36–38], with some advantages, including high throughput, no need for large amounts of DNA, and time and laborsaving [39]. In addition, by using the sequence capture technology on the basis of NGS technology, the genomic DNA fragments containing the insertions were screened out, and then the Illumina sequencing was used to determine the location of the insertion. Inagaki et al. analyzed 29 Arabidopsis transgenic lines and successfully determined the insertion sites of T-DNA in 22 lines, among which 4 lines had multiple insertion sites [16]. Compared with the WGS technology, the sequence capture method was a rapid and cost-effective method for the identification of insertion flanking sequences and the structural characterization of insertion [16]. Because of the libraries of DNA fragment size ranging from 250 bp to 450 bp using in the method and the principle of probes designing, it might be difficult to identifying the flanking sequences and characterizing the structure of exogenous sequences of transgenic lines with two or multiple copies of T-DNA inserted into a locus using the method of Inagaki. On the other hand, NGS combined with targeted bioinformatics analysis has become a sensitive and efficient method for identifying the molecular characteristics of GM crops. Guo et al. used the WGS technology to sequence and analyze the sequence information of two GM soybean events and successfully identified them from a single read analysis [6]. In the work of Siddique et al., the T-DNA insertion site and flanking sequence of the transgenic maize IE09S034 at the 3' end were identified by using NGS and PCR amplification [18]. Although several NGS-based methods have been developed to identify the molecular characteristics of genetically modified crops, some examples often failed to identify insertion sites and flanking sequences in GM crops. For instance, Park et al. used the WGS technology to identify the flanking sequences of three GM rice materials, but one failed to identify the flanking sequences of GM rice [40]. The authors noted that this problem may not arise if they could obtain longer reads. In the present study, we used the WGS method to sequence the transgenic maize lines. After detailed analysis, only one end flanking sequence of the insertion fragments was identified. Generally, the NGS technology used to identify flanking sequences might be efficient if the transgenic line has one or two copies of insertion or stacked transgenic events. On the other hand, the clean reads of the WGS technology are usually only approximately 150 bp, and assembling the flanking sequences requires a large number of reads in the insertion region to be spliced together, which is a considerable challenge for the genome with a large number of repetitive sequences. In our study, ~64.57× coverage of the maize genome was sequenced, and only one end flanking sequence was identified. The insertion sequence consisted of two copies of T-DNA sequences, and it had no further clear sequence information, which increased the difficulty of identifying the flanking sequence using the WGS technology. Increasing the sequence coverage by deep sequencing might be helpful to identify the other end of flanking sequence. However, it is still difficult to characterize the structure of the exogenous sequences using the WGS technology.

The fosmid technology has been applied in genomic studies of many species, such as rice [41], maize [42], and humans [43]. Compared with the BAC library, the construction of the fosmid library is simpler and faster. Furthermore, the average length of the insertion sequences of the fosmid library is 38–48 kb, which might be suitable to identify the flanking sequences and characterize the structure of the insertion sequence of transgenic lines. On the other hand, the read length of single-molecule real-time DNA sequencing might be 10–20 kb, which may also help to characterize the insertion sequence of transgenic events. In our study, three positive clones were accurately identified from the fosmid library using the PCR method with three pairs of specific primers. Furthermore, with the SMRT sequencing technology, the flanking sequences were identified, and the structure of exogenous insertion sequences was characterized. Although the use of the method of building fosmid libraries and third-generation sequencing to obtain flanking sequences of GM crops is more time-consuming and costly than the method based on PCR and WGS, it is more reliable for some GM crops with complex genomic or insertion sequence structures. As a result, when identifying the flanking sequences of genetically modified crops, different methods should be selected flexibly according to their genomic characteristics and the internal structure of insertion sequences (Table 4).

**Table 4:** Characteristic comparison of three methods for obtaining flanking sequences.

Method	Time	Cost	Insertion sequence structure	Genome complexity	Flux level
PCR-based	Short	Cheap	Simple	Simple	Low
NGS	Short	Cheap	Simple	Simple	High
Fosmid + sequencing	Long	Expensive	Complex	Complex	Low

## Supporting information

S1 Fig Vector for transgenic line. (PDF)

S2 Fig Electrophoretogram of fosmid clones digested with *NotⅠ*. 1–16: Insert fragments; M: marker.
(PDF)

S1 Raw images (PDF)
