# Supplementary figures and images for: Identification of genomic insertion and flanking sequences of the transgenic drought-tolerant maize line “SbSNAC1-382” using the single-molecule real-time (SMRT) sequencing method
Source: PLoS One. 2020 Apr 10;15(4):e0226455. doi: 10.1371/journal.pone.0226455 (PMC7147794; doi:10.1371/journal.pone.0226455)

**A**

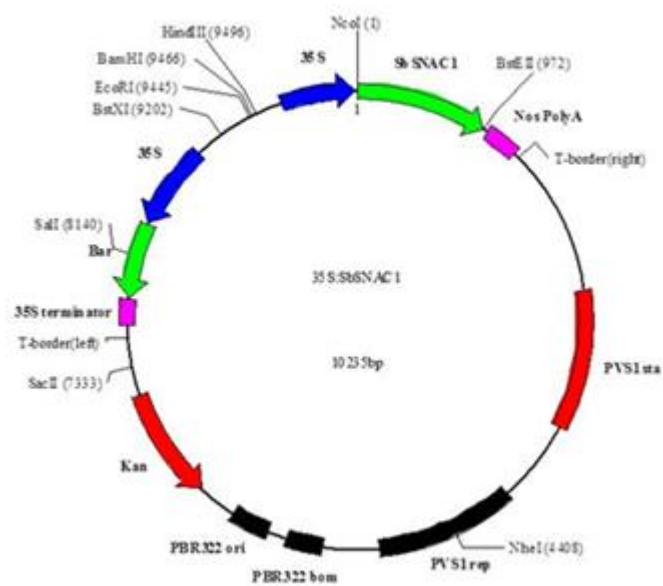

**S1 Figure. Vector for transgenic line.**

Supplement: S1 Fig — (PDF) [file pone.0226455.s001.pdf]

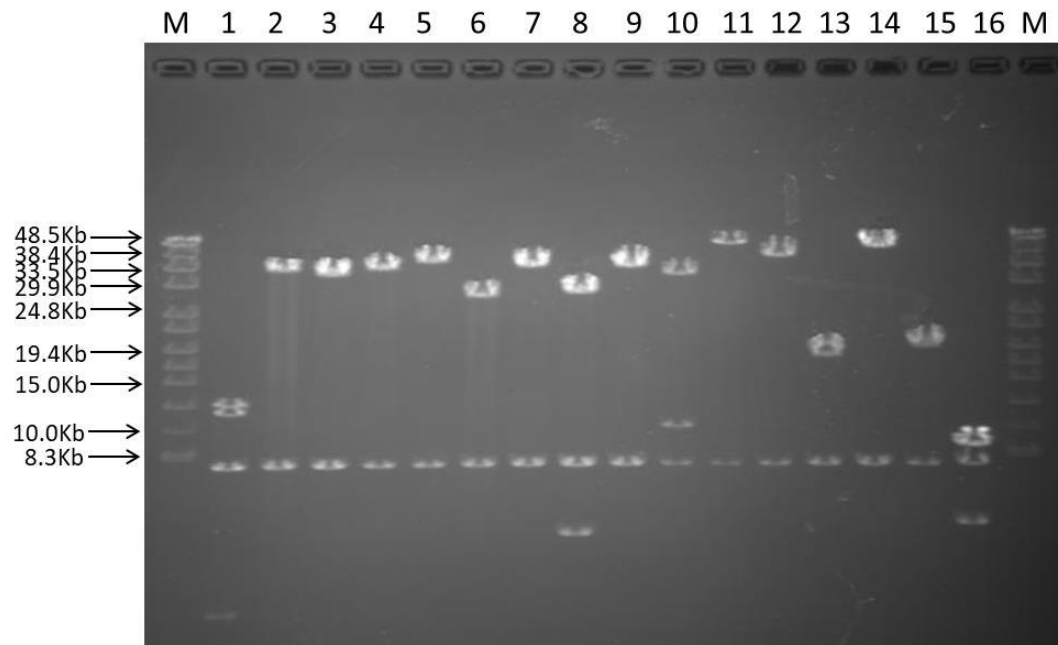

**S2 Figure. Electrophoretogram of fosmid clones digested with *NotI*.** 1-16: Insert fragments; M: Marker.

Supplement: S2 Fig — 1–16: Insert fragments; M: marker. (PDF) [file pone.0226455.s002.pdf]
