# Supplementary material for: Identification of genomic insertion and flanking sequences of the transgenic drought-tolerant maize line “SbSNAC1-382” using the single-molecule real-time (SMRT) sequencing method
Source: PLoS One. 2020 Apr 10;15(4):e0226455. doi: 10.1371/journal.pone.0226455 (PMC7147794; doi:10.1371/journal.pone.0226455)
Supplement: S1 Raw images — (PDF) [file pone.0226455.s003.pdf]

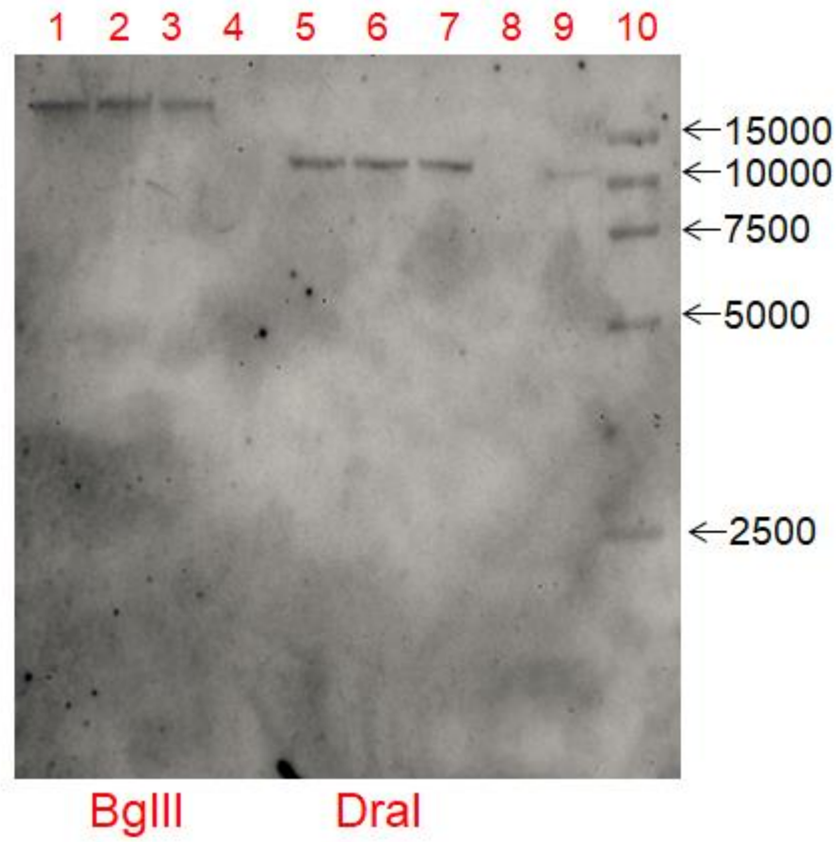

Figure 1 Southern blot of transgenic line 382 with bar probe. Digested DNA of the transgenic line 382 by BglII and DraI. Lanes 1 to 10: 1:16HT382; 2:16BJT382; 3:15HT382; 4:zheng58; 5:16HT382; 6:16BJT382; 7:15HT382; 8:zheng58; 9: digested plasmids as positive controls; 10: marker.

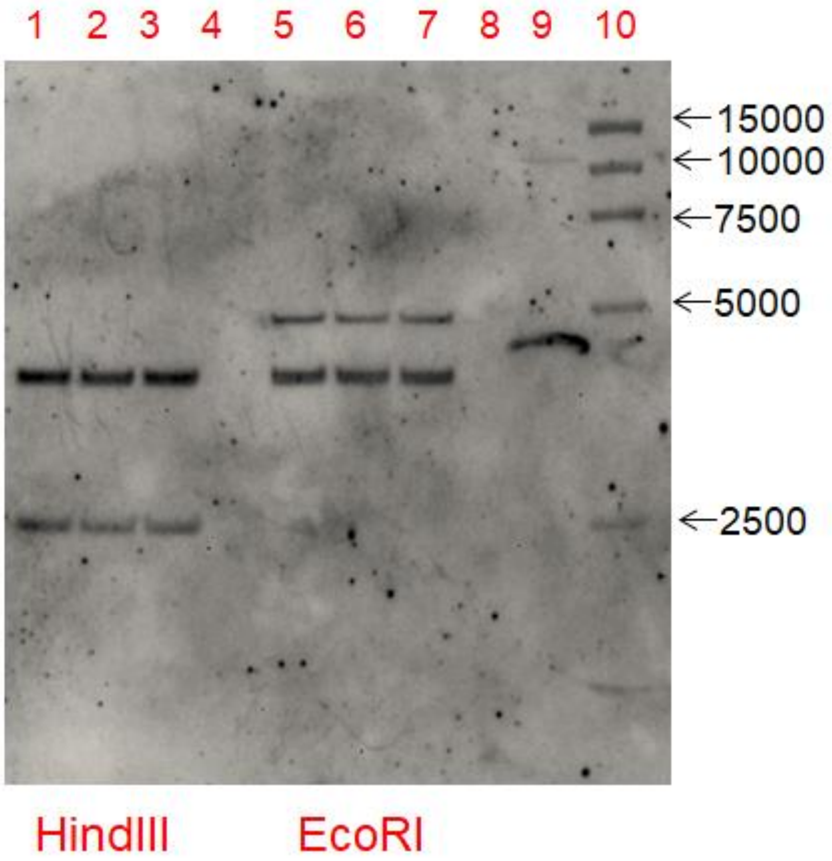

Figure 2 Southern blot of transgenic line 382 with bar probe. Digested DNA of the transgenic line 382 by HindIII and EcoRI. Lanes 1 to 10: 1:16HT382; 2:16BJT382; 3:15HT382; 4:zheng58; 5:16HT382; 6:16BJT382; 7:15HT382; 8:zheng58; 9: digested plasmids as positive controls; 10: marker.

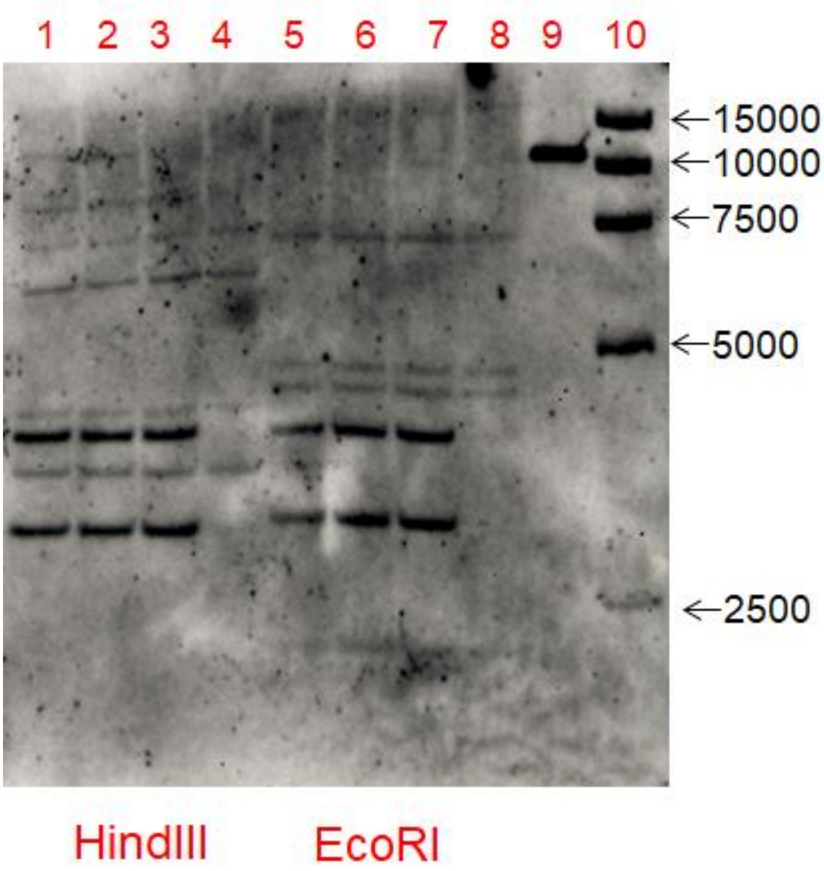

Figure 3 Southern blot of transgenic line 382 with SbSNAC1 probe. Digested DNA of the transgenic line 382 by HindIII and EcoRI. Lanes 1 to 10: 1:16HT382; 2:16BJT382; 3:15HT382; 4:zheng58; 5:16HT382; 6:16BJT382; 7:15HT382; 8:zheng58; 9: digested plasmids as positive controls; 10: marker.

Figure 1 in the manuscript consists of the three pictures above. All Southern blots were obtained in 1.5% agarose gel, 30V, and electrophoresis for 16h. Because the safety assessing of transgenes requires the Southern blot of materials for three consecutive years (16HT382; 16BJT382; 15HT382), we have integrated the information of these three images into a figure to illustrate the problem. IN the figure3, because of the high homology between *SbSNAC1* and *ZmNAC1* gene in the background of maize, there will be weak bands in the hybridization of SbSNAC1 probe in maize transgenic line 382, but the bands of the target gene are strong. In the manuscript, the six lanes in Figure 1 are composed of lanes 3 and 7 in Figure 2, lanes 3 and 6 in Figure 1, and lanes 9 and 10 in Figure 3.

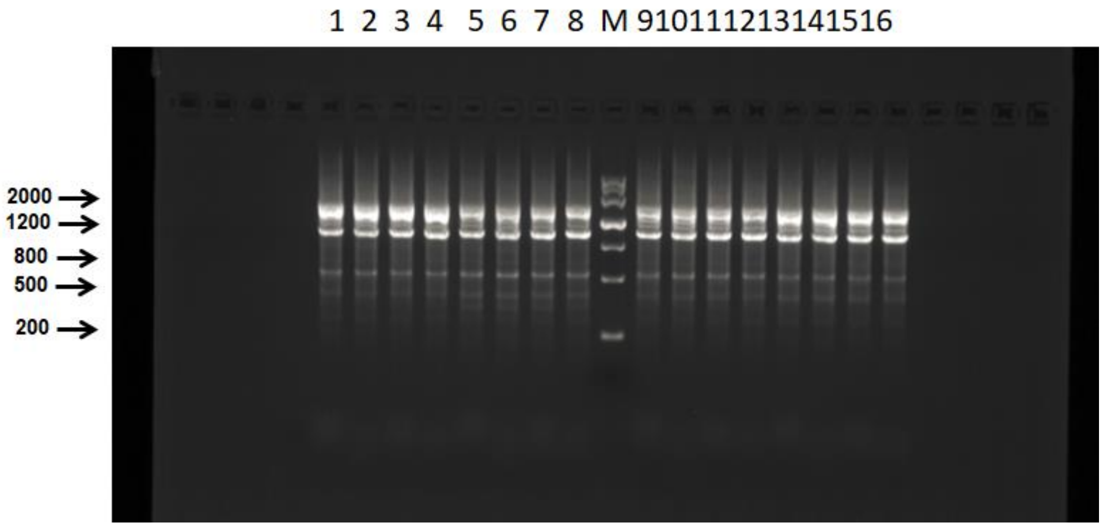

Figure 4 Genome walking results for 5’ flanking sequence of four materials. Lane1-4 are the first amplification results of transgenic line 463 of specific primer zsp1 and degenerate primer AP1-AP4, respectively; Lane5-8 are the first amplification results of transgenic line 389 of specific primer zsp1 and degenerate primer AP1-AP4, respectively; Lane9-12 are the first amplification results of transgenic line 382 of specific primer zsp1 and degenerate primer AP1-AP4, respectively; Lane13-16 are the first amplification results of transgenic line 380 of specific primer zsp1 and degenerate primer AP1-AP4, respectively.

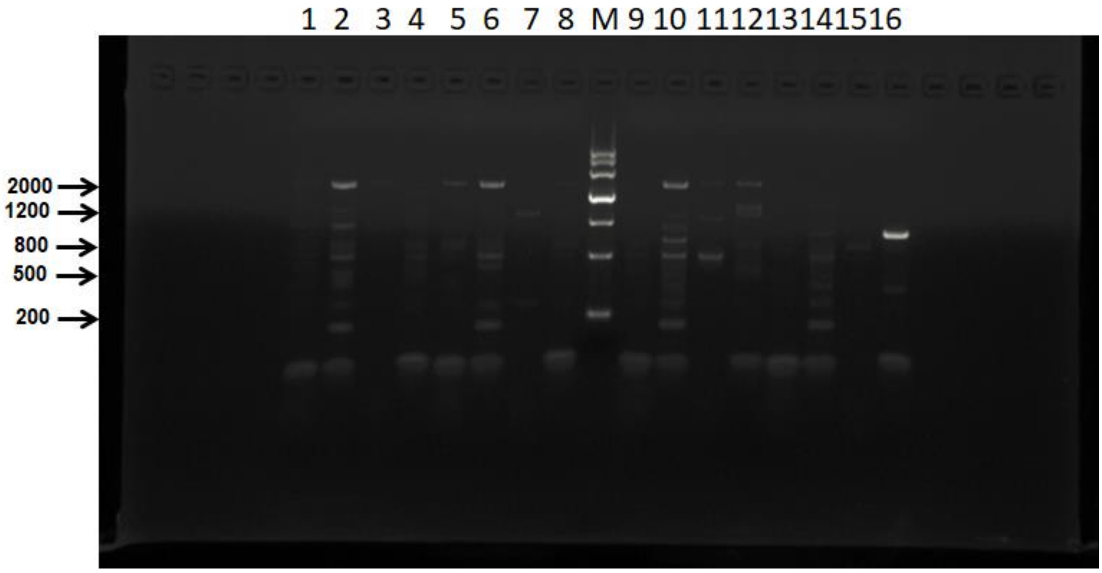

Figure 5 Genome walking results for 5’ flanking sequence of four materials. Lane1-4 are the second amplification results of transgenic line 463 of specific primer zsp1 and degenerate primer AP1-AP4, respectively; Lane5-8 are the second amplification results of transgenic line 389 of specific primer zsp1 and degenerate primer AP1-AP4, respectively; Lane9-12 are the second amplification results of transgenic line 382 of specific primer zsp1 and degenerate primer AP1-AP4, respectively; Lane13-16 are the second amplification results of transgenic line 380 of specific primer zsp1 and degenerate primer AP1-AP4, respectively.

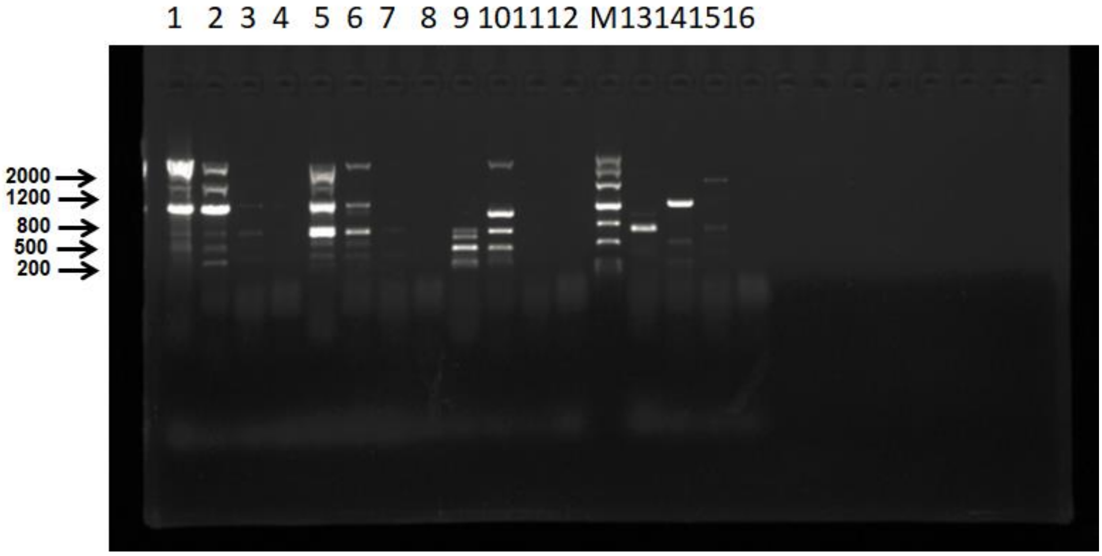

Figure 6 Genome walking results for 5' flanking sequence of four materials. Lane1-4 are the third amplification results of transgenic line 463 of specific primer zsp1 and degenerate primer AP1-AP4, respectively; Lane5-8 are the third amplification results of transgenic line 389 of specific primer zsp1 and degenerate primer AP1-AP4, respectively; Lane9-12 are the third amplification results of transgenic line 380 of specific primer zsp1 and degenerate primer AP1-AP4, respectively; Lane13-16 are the third amplification results of transgenic line 382 of specific primer zsp1 and degenerate primer AP1-AP4, respectively.

Figure 2 in the manuscript consists of lane 9-12 of figure 4, lane 9-12 of figure 5 and lane 13-16 of figure 6. The above three pictures were all in 1% agarose gel, 120 V, electrophoresis for 30mins.

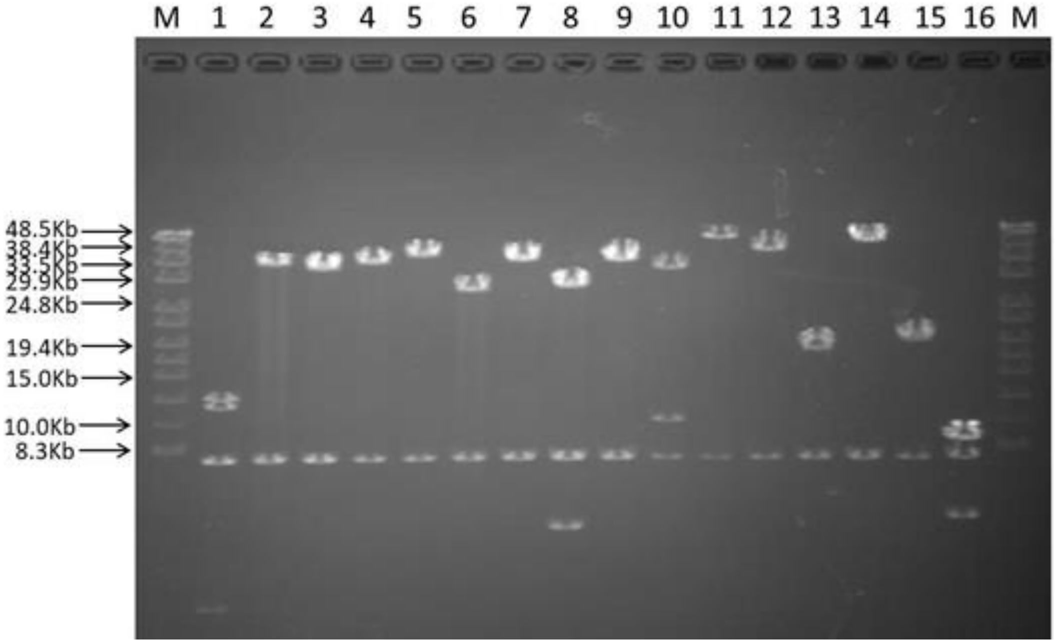

Figure 7 Electrophoretogram of fosmid clones digested with Not Ⅰ. 1-16: Insert fragments; M: Marker.

Figure S2 in the supplementary material is figure 7, which is the original figure.

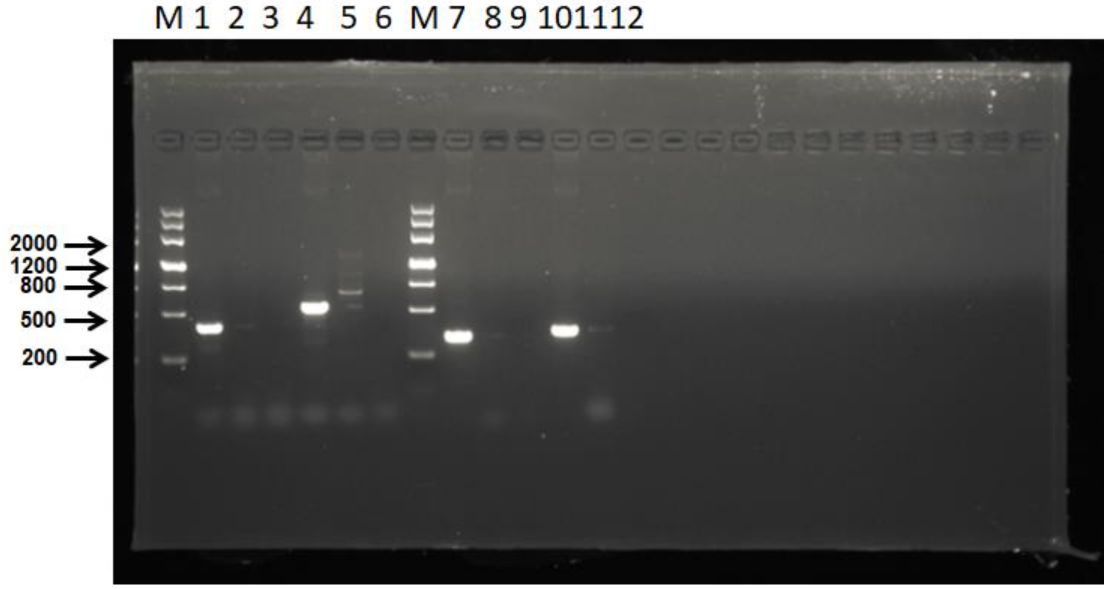

Figure 8 PCR validation of transgenic insertion sites. Lane 1-6, PCR verification of 3'end of inserted sequence. Lane 1, 2, 3 and 4, 5, 6 primer V1/G1 and V1/G2 amplified in transgenic line, negative control zheng58, negative control of water, respectively. Lane 7-12, PCR verification of 5'end of inserted sequence. Lane 7, 8, 9 and 10, 11, 12 primer YZP1/YZP2 and YZP1/YZP3 amplified in transgenic line, negative control zheng58, negative control of water, respectively. M: marker.

Figure 3 in the manuscript consists of lane 7-12 and lane 1-6 of the figure 8. The above picture was in 1% agarose gel, 120 V, electrophoresis for 30mins.
